# High-Throughput Determination of Major Mycotoxins with Human Health Concerns in Urine by LC-Q TOF MS and Its Application to an Exposure Study

**DOI:** 10.3390/toxins14010042

**Published:** 2022-01-05

**Authors:** Noelia Pallarés, Dionisia Carballo, Emilia Ferrer, Yelko Rodríguez-Carrasco, Houda Berrada

**Affiliations:** 1 Department of Preventive Medicine and Public Health, Food Science, Toxicology and Forensic Medicine, Faculty of Pharmacy, University of Valencia, 46100 Burjassot, Spain; noelia.pallares@uv.es (N.P.); houda.berrada@uv.es (H.B.); 2 Faculty of Agricultural Science, National University of Asunción, San Lorenzo 2160, Paraguay; dionisia.carballo@agr.una.py

**Keywords:** biomarkers, mycotoxins, QuEChERS, LC-ESI-QTOF, urine, risk assessment

## Abstract

Human biomonitoring constitutes a suitable tool to assess exposure to toxins overcoming the disadvantages of traditional methods. Urine constitutes an accessible biological matrix in biomonitoring studies. Mycotoxins are secondary metabolites produced naturally by filamentous fungi that produce a wide range of adverse health effects. Thus, the determination of urinary mycotoxin levels is a useful tool for assessing the individual exposure to these food contaminants. In this study, a suitable methodology has been developed to evaluate the presence of aflatoxin B2 (AFB2), aflatoxin (AFG2), ochratoxin A (OTA), ochratoxin B (OTB), zearalenone (ZEA), and α-zearalenol (α-ZOL) in urine samples as exposure biomarkers. For this purpose, different extraction procedures, namely, the Solid Phase Extraction (SPE); Dispersive Liquid–Liquid Microextraction (DLLME); and Quick, Easy, Cheap, Effective, Rugged, and Safe (QuEChERS) methods were assessed, followed by Liquid Chromatography coupled to Quadrupole Time of Flight Mass Spectrometry with Electrospray Ionization (LC-ESI-QTOF-MS) determination. Then, the proposed methodology was applied to determine mycotoxin concentrations in 56 human urine samples from volunteers and to estimate the potential risk of exposure. The results obtained revealed that 55% of human urine samples analyzed resulted positive for at least one mycotoxin. Among all studied mycotoxins, only AFB2, AFG2, and OTB were detected with incidences of 32, 41, and 9%, respectively, and levels in the range from <LOQ to 69.42 µg/L. Risk assessment revealed a potential health risk, obtaining MoE values < 10,000. However, it should be highlighted that few samples were contaminated, and that more data about mycotoxin excretion rates and their BMDL10 values are needed for a more accurate risk assessment.

## 1. Introduction

Traditional evaluation exposure to mycotoxins is often carried out combining the analysis of chemicals in foodstuffs with food consumption data. However, this indirect approach presents some disadvantages such as the lack of information related to the individual exposure situation, toxicokinetics, and bioavailability of the selected food contaminants [1]. Furthermore, this approach presents the difficulty of obtaining accurate data on food consumption and the bioavailability of toxins. The distribution of mycotoxin levels in food is not homogeneous and some mycotoxins may be linked to food matrix components and may, therefore, be underestimated [2].

Human biomonitoring constitutes a suitable alternative in order to assess toxin exposure at individual level and has already been applied to study exposure to mycotoxins in different countries and cohorts. The typical biomarkers used for exposure assessment are the parent toxins and the major phase I and II metabolites. Blood, urine, or breast milk samples are the biological fluids most often used in biomonitoring. Among them, the use of non-invasive urine sampling is the most frequent [3,4,5].

Mycotoxins are low-molecular weight secondary metabolites produced by fungi during preharvest, harvest, or storage steps. These toxic compounds are produced to a response of oxidative stress during fungi colonization and infestation. *Aspergillus*, *Fusarium,* and *Penicillium*, are the major mycotoxin producers [6]. Although more than 300 mycotoxins have been identified, only some of them have been regulated in food by the European Commission (EC 1881/2006) [7]. Aflatoxins (AFs) are produced by species of *Aspergillus* genera; ochratoxin A (OTA) is produced by both *Aspergillus* and *Penicillium*; trichothecenes (HT-2, T-2, deoxynivalenol (DON) as well as nivalenol (NIV)), zearalenone (ZEA), fumonisins (FB1 and FB2) and emerging mycotoxins are produced by *Fusarium* species. Aflatoxins (AFs), ochratoxin A (OTA), zearalenone (ZEA), trichothecenes, fumonisins (FBs), and patulin (PAT) constitute the mycotoxins most likely to occur in foodstuffs [8,9].

Human exposure to mycotoxins occurs through the consumption of contaminated crops or derivatives or through the ingestion of animal origin products from animals fed with contaminated feed. Humans can also be exposed to mycotoxins by inhalation and dermal contact. The impact of mycotoxins on human health depends on different factors such as the type of toxin, its metabolism, the pharmacokinetics, the exposure conditions, and the health status of the individual [2]. Long-term exposure to high doses can produce health problems such as mutagenicity, carcinogenicity, teratogenicity, hepatotoxicity, nephrotoxicity, gastrointestinal toxicity, immunotoxicity, and neurotoxicity [10].

Mycotoxins can occur in three possible forms: unmodified as they are biosynthesized by fungal metabolisms (basic or free forms of mycotoxin structures); matrix-associated as complexes with matrix compounds; modified mycotoxins that have undergone chemical or biological modifications to their structure. These modifications of mycotoxin structures can be produced by fungi, plants, or animals that are able to modify toxins because of their metabolic processes. Modified mycotoxins can be converted to free toxin forms during digestion, thus increasing exposure to these toxins [11]. All mycotoxin forms (free, metabolites, and conjugates) should be included in biomonitoring studies for a more realistic approach of risk assessment [12].

This study focuses on the presence of the major mycotoxins with human health concerns: AFs, OTA, and ZEA.

AFs are very toxic compounds classified as carcinogenic to humans by the International Agency for Research in Cancer (IARC) [13]. O-dealkylation, ketoreduction, epoxidation, and hydroxylation constitute the AFB1 major metabolic pathways. These reactions lead to the production of highly toxic forms, such as AFB1-8,9-epoxide (AFBO) and aflatoxin M1 (AFM1), as well as relatively nontoxic forms: aflatoxin P1 (AFP1), aflatoxin Q1 (AFQ1), or aflatoxin 2a (AFB2a) [14].

OTA constitutes a toxic compound with relatively rapid absorption and slow elimination. The liver and kidneys are the main organs involved in OTA biotransformation. OTA has affinity to plasma proteins, approximately 99% of the circulating OTA is bound to plasma proteins. OTA is eliminated both via urine and feces after biliary excretion [15]. The major OTA metabolic pathways are hydrolysis, hydroxylation, lactone-opening, and conjugation. OTalpha (OTα) constitutes the major OTA metabolite and is formed by the cleavage of the peptidic bond. OTA can also be metabolized into its hydroxylated derivatives: 4-R-hydroxyochratoxine A (4-R-OH OTA), 4-S-hydroxyochratoxine A (4-S-OH OTA), 10-hydroxyochratoxin A (10 OH-OTA), ochratoxin B (OTB), open lactone of ochratoxin (OP-OTA) and ochratoxin hydroquinone (OTHQ), which can be found in blood or urine in the cited forms or conjugated to glutathione. Most of the OTA metabolites such as OTα and OTB are considered less toxic than the parent compound [16].

ZEA presents a hormonal action higher than others naturally occurring non-steroidal estrogens due to its structural similarity to 17β-estradiol. ZEA is mainly metabolized in the liver and intestine in different ways: reduction reactions, resulting in α-zearalenol (α-ZOL), β-zearalenol (β–ZOL), α-zearalanol (α-ZAL), β-zearalanol (β-ZAL) and zearalanone (ZAL) metabolites, monohydroxylation producing ZEA catechols, and a conjugation reaction that implies the conjugation of ZEA and its reduced metabolites with sulfate and glucuronic acid. α-ZEA presents more estrogenic potential than the ZEA parent compound [17,18].

Urine samples constitute a complex matrix, in which mycotoxins presented in trace amounts may be masked by some interfering compounds, making a clean-up step necessary. Different techniques such as liquid–liquid extraction (LLE), solid phase extraction (SPE), dispersive liquid–liquid micro extraction (DLLME), QuEChERS, and immunoaffinity columns (IAC) have been reported in the literature for mycotoxin determination in urine [19,20,21,22].

Previous enzymatic treatment of urine samples with β-glucuronidase is required to release the parent mycotoxin from the mycotoxins–glucuronides. After enzymatic deconjugation, the total amount of (free +conjugated) mycotoxins can be measured.

The aim of the present study was to develop a suitable methodology to assess human exposure to mycotoxins (AFB2, AFG2, OTA, OTB, ZEA, and α-ZOL) through biomonitoring analysis. For this purpose, different extraction procedures (SPE, DLLME, QuEChERS) were evaluated with determination by Liquid Chromatography coupled to Quadrupole Time of Flight Mass Spectrometry with an Electrospray Ionization (LC-ESI-QTOF-MS) system. Then, the proposed procedure was validated and applied to determine mycotoxin concentrations in 56 human urine samples from volunteers.

## 2. Results

### 2.1. Evaluation of SPE, DLLME, and QuEChERS Extraction Methods

**Figure 1:**
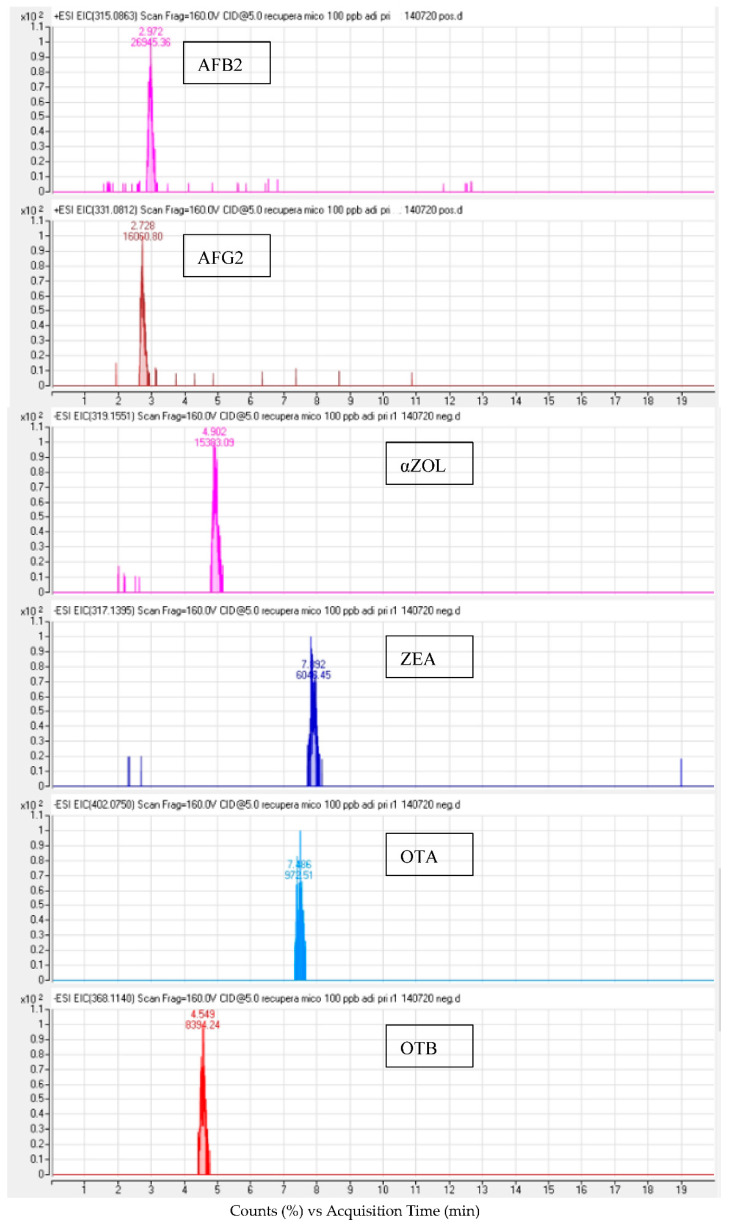
LC-ESI-QTOF chromatogram of a spiked urine sample at 100 µg/L after QuEChERS extraction.

QuEChERS extraction was selected as the most appropriate methodology, as it provided better recovery values for the mycotoxins studied at both recovery levels, from 55 to 90% at 50 µg/L and from 75 to 93% at 100 µg/L (Figure 1). Lower recovery values ranging from 7 to 30% were obtained with the SPE method, being OTA the mycotoxin with lowest recovery percentage achieved (7%). The same trend was observed also for OTA employing DLLME and QuEChERS methods. Finally, DLLME extraction provided at 100 µg/L, recovery values under 30% for OTA and ZEA, 51% for AFG2, and 68% for AFB2, and near to 100% for OTB and α-ZOL. As QuEChERS extraction revealed adequate recovery percentages for all studied mycotoxins at the two levels of concentration assayed, it was proposed as the most appropriate for further validation.

In a previous mycotoxin extraction comparative study, where was optimized the AFB1, AFB2, AFG1, AFG2, OTA, ZEA, beauvericin (BEA), enniatins (ENNs) extraction from urine samples, QuEChERS extraction showed similar recovery percentages (71–109%); however, DLLME was selected by these authors as it provided slightly better values (79–113%) [20]. Contrary to the present study, better extraction efficiency values (70–98%) were obtained employing SPE cartridges for NIV, DON, Deepoxy-deoxynivalenol, Aflatoxin M1, FB1, Dihydrocitrinone, Alternariol, Citrinine, α-ZOL, β-ZOL, OTA, and ZEA determination in urine. The different results obtained in the present study may be due the different type of cartridge used, and the different alternatives of solvent employed for the elution and reconstitution steps [23].

### 2.2. Validation of the QuEChERS Method

All analytical parameters obtained (recoveries, matrix effects, limits of detection, and quantification and linearity) were in accordance with the limits established by European Commission Decision 2002/657/EC [24] (Table 1).

Recoveries obtained ranged from 52 to 115% at 50 μg/L and from 68 to 105% at 100 μg/L, respectively.

SSE (%) obtained evidenced a signal suppression (less than 50%) for all mycotoxins except for AFB2 and AFG2. Therefore, matrix-matched calibration curves were used to compensate the signal suppression effects and for effective quantification of the samples. Matrix-matched calibration curves were constructed by spiking blank urine extract samples at levels between <LOQ and 1000 μg/L.

Calibrations curves revealed good linearity, with correlation coefficients (R^2^) between 0.990 and 0.999.

Finally, LOD values ranged from 1.5 to 5 µg/L, while LOQ ranged from 3 to 10 µg/L.

### 2.3. Mycotoxin Biomarker Occurrence in Urine Samples

About 55% of the 56 human urine samples resulted positive for at least one of the studied mycotoxins. Comparing genders, 15 of (*n* = 24) male urine samples were contaminated, while only 16 of (*n* = 32) female urine samples were positive. Regarding the analyzed mycotoxins, only AFB2, AFG2, and OTB were detected in hydrolyzed human urine samples with incidences of 32, 41, and 9%, respectively, and concentrations ranging from <LOQ to 69.42 µg/L (Table 2).

AFG2 turned out to be the most prevalent mycotoxin, being reported in 41% of total samples with contents, ranging from <LOQ to 69.42 µg/L. The mean of positive samples was 23.81 µg/L. Comparing genders, 54% of male urine samples resulted contaminated with AFG2, against 31% of female samples. No significant differences were found between the mean of positive samples in both genders, with 24.97 µg/L for males and 22.28 µg/L for females, respectively. AFB2 was reported in 32% of total urine samples at levels comprised between <LOQ and 60.98 µg/L, and a mean of positive samples of 16.48 µg/L. A similar mean concentration was observed when male and female urine samples were compared (19.16 µg/L and 14.78 µg/L, respectively). Similar results were obtained by Jonsyn-Ellis [25], who analyzed mycotoxin levels in urine samples from 97 boys and 93 girls from Sierra Leone during the rainy season. These authors reported AFB1 in 33% and 41% of the boys’ and girls’ samples, respectively, at concentrations ranging from 0.08 to 127 µg/L. AFB2 was detected in 9% and 20% of the boys’ and girls’ samples, respectively, with levels of up to 48 µg/L, while AFG1 was detected in 28 and 19% of samples, respectively, at concentrations of up to 57.4 µg/L in boys and 150 µg/L in girls. Finally, AFG2 was only detected in 2 and 3% of samples, respectively, at low concentrations (≤2 µg/L). AFB2 was reported by these authors at similar concentrations to those observed in the present study: although, AFG2 was detected at lower concentrations than in the present study.

Slightly lower concentrations were reported by Ritieni et al. [26] in Italy after analyzing 18 urine samples from pregnant women. AFG1 was reported by these authors in four samples at concentrations ranging from 14.0 to 18.8 µg/L, while AFB1 and AFB2 were presented in two samples at concentrations of 0.4–2 µg/L and 0.3–3 µg/L, respectively. Contrary to these results, in a study performed in Brazil, Jager et al. [27] did not detect AFs in any of the 16 analyzed urine samples, while AFM1 was reported in 61% of the samples. Similar to these authors, Rubert et al. [28] did not report the presence of AFB1, AFB2, and AFG1 in any urine samples acquired from 27 volunteers, while AFG2 was detected only in one sample at trace levels, at a concentration value comprised between LOD (0.8 µg/L) and LOQ (2 µg/L).

Regarding ochratoxins, OTA was not detected in any of the analyzed samples. Concerning the information available in the literature about the presence of OTA in urine samples, similarly to the present study, Ritieni et al. [26] did not report OTA in any of the 18 urine samples from pregnant Italian women. Rubert et al. [28] only reported the presence of OTA at trace level in 3 of 27 urine samples in a study performed in Spain. Contrary to these results, also in a Spanish population, Coronel et al. [29] reported OTA and ochratoxin α (OTα) presence in 72 human urines samples enzymatically treated, with an occurrence of 12.5% and 61.1%, respectively, and concentrations of up to 0.562 ng/mL and 2.894 ng/mL, respectively. In Portugal, Martins et al. [21] revealed the exposure of the Portuguese population to OTA, among other mycotoxins. However, OTA was observed in first-morning urine samples only at levels of up to 0.610 μg/L. Higher contents and incidences were also reported by Jonsyn-Ellis [25], who studied the presence of aflatoxins and ochratoxins in children’s urine samples from Sierra Leone. In the urine samples collected during the dry season, OTA was detected in 21% of the boys’ samples at contents ranging between 0.07 and 59 ng/mL, while in girls an occurrence of 31% was detected and contents in the range of 0.08–148 ng/mL. The contents detected in the urine collected in the rainy season ranged from 0.6 to 72.2 ng/mL for boys and from 0.7 to 4.9 ng/mL for girls, respectively.

In contrast to OTA, the other ochratoxin studied in the present work, OTB, was observed in 9% of the urine samples with concentrations ranging from <LOQ and 38.88 µg/L, and a mean of positives of 18.17 µg/L. The mean amount detected in male samples (38.88 µg/L) was higher than that detected in females (12.99 µg/L). Similar to the present study, Jonsyn-Ellis [25] reported some of the urine samples from boys and girls from Sierra Leone positive for OTB. During the rainy season, ranges between 0.05 and 45 ng/mL and between 0.06 and 81 ng/mL were observed for boys and girls, respectively. Similar concentrations were reported in the present study, although a higher OTB occurrence (up to 44%) was reported by Jonsyn-Ellis [25]. In contrast, Liu et al. [30] did not detect OTB in any of the 60 human urine samples collected in Beijing (China) after enzymatic digestion with β-glucuronidase. Contrary to OTB, OTA, and OT-alpha were detected by these authors in one-third of samples at concentrations of up to 0.14 and 2.38 ng/mL, respectively.

ZEA and its metabolite α-ZOL, were not detected in any of the analyzed urine samples. Similar to the present study, Solfrizzo et al. [31] did not report α-ZOL presence in 10 human urine samples after enzymatic pre-treatment of urine with β-glucuronidase/sulfatase. Contrary to the present work, in China, Li et al. [32] analyzed 301 urine samples collected from volunteers aged 0–84 with and without enzyme hydrolysis to determine total and free ZEA biomarkers (α-ZOL, β-ZOL, α-ZAL, β-ZAL, and ZAN). ZEA and α-ZOL were reported by these authors at incidences of 71 and 4%, respectively, after enzyme hydrolysis and concentrations ranging from <LOQ and 3.7 µg/L. Further, in a Chinese population, Zhang et al. [33] studied human biomonitoring of ZEA and its metabolites (α-ZOL, β-ZOL, ZAN, α-ZAL, and β-ZAL) in 199 urine samples, both free and total after β-glucuronidase digestion. After enzymatic hydrolysis, total ZEN, α-ZEL, and β-ZEL were detected in 87.8, 25.6, and 24.1% of samples, respectively, with average amounts of 0.383 ng/mL, 0.089 ng/mL, and 0.142 ng/mL, respectively. These authors also observed that positive rates and amounts increased after enzymatic hydrolysis.

### 2.4. Mycotoxin Biomarker Risk Assessment

For AFB2, a PDI of up to 28.3 µg/kg bw/day was calculated considering the mean of positives samples, decreasing to values of 9.1 µg/kg bw/day and 10.85 µg/kg bw/day under the LB and UB scenarios, respectively (Table 3). Regarding AFG2, a PDI of up to 40.9 µg/kg bw/day was calculated, decreasing to 15.9 µg/kg bw/day (LB scenario) and 17.2 µg/kg bw/day (UB scenario). Similar to the present study, Martins et al. [34] assessed the Portuguese exposure to aflatoxins employing a biomonitoring approach in urine and obtained AFs PDIs of 13.4 and 16.7 µg/kg bw/day considering the probabilistic and deterministic approaches. However, all PDI obtained in the present study estimated a MoE < 10,000, thus revealing a potential health risk.

Concerning OTB, PDI values of up to 0.81, 0.07, and 0.19 µg/kg bw/day, were calculated within mean of positive samples estimation, LB and UB scenarios, respectively (Table 3). These PDIs exceeded the TWI established for OTA in all scenarios. In Catalonia (Spain), Vidal et al. [35] reported a median OTA PDI of 0.031 µg/kg bw/day after the analysis of urine samples, with most samples exceeding the safety TWI value fixed for OTA as in the present study. These authors also concluded that the dietary exposure approaches may result in an underestimation of mycotoxin exposure. Moreover, the margin of exposure (MoE) obtained using the BMDL10 established for OTA also revealed a potential health risk with values close to 100 and 200 in LB and UB scenarios. However, several studies revealed that OTB is metabolized and eliminated quickly and completely, unlike OTA, presenting a lower nephrotoxicity. Furthermore, in vitro and in vivo studies suggested that OTB could be approximately an order of magnitude less toxic than OTA [36]. Thus, much more accurate risk assessment could be performed with the OTB excretion rate and BMDL10 specific values.

## 3. Conclusions

Among all extraction procedures evaluated (SPE, DLLME and QuEChERS), QuEChERS extraction followed by LC-ESI-qTOF determination was selected as the most appropriate methodology to determine AFB2, AFG2, OTA, OTB, ZEA, and α-ZOL biomarkers in urine samples, as it produces better recovery results (55–93%). The remainder of the analytical parameters obtained was also in accordance with the limits established by European Commission Decision 2002/657/EC. The application to actual urine samples from volunteers revealed the presence of AFB2, AFG2, and OTB in 32, 41, and 9% of the analyzed samples at levels ranging from <LOQ to 69,42 µg/L. Risk assessment revealed a potential health risk to AFB2, AFG2, and OTB exposure. However, it is important to highlight that only some samples were contaminated and that more data about mycotoxin excretion rates and BMDL10 values are needed to obtain a more accurate risk assessment.

## 4. Materials and Methods

### 4.1. Reagents and Chemicals

Solvents, acetonitrile (ACN) and methanol (MeOH) TOF grade and ethyl acetate (EtOAc) were supplied by Merck (Darmstadt, Germany). Deionized water (<18.2 MΩcm resistivity) was prepared in the laboratory using a Milli-QSP^®^ Reactive Water System (Millipore, Beadford, MA, USA). Formic acid (CH_2_O_2_) (grade ≥ 95%) was supplied by Sigma-Aldrich (St. Louis, MO, USA), and acetic acid (C_2_H_4_O_2_) (grade ≥ 99%) was acquired from Fisher Scientific (UK). All solvents were filtered through a 0.45 µm cellulose filter supplied by Scharlau (Barcelona, Spain).

Salts, ammonium formate (99%) was supplied by Panreac Quimica S.A.U (Barcelona, Spain), sodium chloride (NaCl) was obtained from VWR Chemicals (Leuven, Belgium), magnesium sulfate (MgSO4) anhydrous powder (99.5%) was supplied by Alfa Aesar (Karlsruhe, Germany) and C18-E (50 µm, 65 A) was purchased from Phenomenex (Madrid, Spain).

Cartridges used for SPE extraction consisted in Strata 33 μm polymeric reversed phase supplied by Phenomenex (USA).

Ammonium acetate was obtained from Merck. Helix pomatia type H-1 β-glucuronidase (glucuronidase activity: ≥300,000 units/g solid and sulfatase activity: ≥10,000 units/g solid) was purchased from Sigma-Aldrich.

Mycotoxin standards (OTA, OTB, AFB2, AFG2, ZEA, and α-ZOL) were obtained from Sigma Aldrich. Individual stocks solutions of all analytes were prepared to obtain 100 mg/L in methanol, and 1 mg/L multi-analyte working solutions were prepared by diluting the individual solutions. All standards were stored in the dark at −20 °C.

Before the injection, samples were filtered through a nylon syringe filter (13 mm diameter, 0.22 µm pore size) obtained from Membrane Solutions (Texas TX, USA).

### 4.2. Sample Collection

Fifty-six urine samples from adult participants were collected during the December 2019–January 2020 period. First-morning urine samples collected were obtained from 24 males and 32 females in a wide age group. To collect the samples, sterile vessels were used and then stored at −20°C until analysis. No exclusion criteria were set, and volunteers provided a signed informed consent following the Helsinki Declaration on ethical principles for medical research. This research was approved by the University of Valencia Institutional Human Research Committee (reference number: 1564214).

### 4.3. Urine Sample Preparation

All urine samples were centrifuged at 5000 rpm for 5 min at 4 °C prior to extraction.

The urine samples were hydrolyzed according to a previous study [5]: 1 mL of the previously centrifuged urine was collected in a 2 mL Eppendorf tube and 250 µL of ammonium acetate buffer (1 M, pH5) containing 20,000 U of β-glucuronidase/mL was added. Hydrolysis was performed with continuous stirring at 550 rpm for 18 h at 37 °C.

### 4.4. Mycotoxin Extraction Procedures

#### 4.4.1. Solid Phase Extraction (SPE)

Firstly, 1 mL of previously centrifuged urine was introduced into the cartridges pre-conditioned with 1 mL of MeOH and 1 mL of H_2_O. Samples were eluted from the cartridges using 600 µL of 2% formic acid MeOH/ACN (50:50). Then, the samples were dried under a nitrogen stream and reconstituted with 1 mL of 0.1% formic acid ACN/MeOH.

#### 4.4.2. Dispersive Liquid–Liquid Microextraction (DLLME)

For DLLME extraction, 1 mL of previously centrifuged urine was placed in a tube with 0.3 g of NaCl and mixed with the vortex, then 1 mL of ACN and 100 µL of EtOAc were added and vortexed for 1 additional minute. Thereafter, the samples were centrifuged at 5000 rpm at 4 °C for 3 min, the organic phase, separated and placed at the top of the tube, was collected. Then, it was evaporated under a N_2_ stream and reconstituted with 1 mL MeOH /H_2_O (70:30, *v*/*v*) prior to being filtered through a 13 mm/0.22 μm nylon filter.

#### 4.4.3. QuEChERS

Firstly, 1 mL of the previously centrifuged urine was placed into a 15 mL tube with 0.3 g of MgSO4, 0.030 g of C18 and 1 mL of ACN. After vortex for 1 min and centrifugation at 4500 rpm for 3 min, the supernatant was then filtered through a 13 mm/0.22 μm nylon filter inside 1.5 mL glass vials prior to injection.

#### 4.4.4. Optimization of Extraction Procedures

To optimize the extraction of AFB2, AFG2, OTA, OTB, ZEA, and α-ZOL mycotoxins from urine samples different extraction protocols were tested: SPE, DLLME, and QuEChERS.

Optimization was carried out through recovery experiments. For this, the absolute peak areas of each analyte in a blank urine sample spiked before extraction was compared with the absolute peak areas of the analyte spiked after extraction. Recovery experiments were performed in triplicate at two levels of contamination (50 μg/L and 100 μg/L).

#### 4.4.5. Validation of the QuEChERS Method

QuEChERS method was characterized in terms of recoveries, linearity, limits of detection (LODs), and limits of quantification (LOQs) and matrix effects according to European directive 2002/657/EC [24].

Recovery experiments were performed at two levels (50 and 100 μg/L). Intra-day analysis was obtained by three determinations on the same day, and inter-day was assessed based on three determinations on nonconsecutive days.

To evaluate possible matrix effects, which evidence a possible suppression or enhancement of mycotoxin signals, the slope of mycotoxin calibration curves prepared in blank urine extract samples were compared with the slope of calibration curves performed in solvent. SSE (%) were calculated as follows: SSE (%) = slope of curve prepared in extracted matrix/slope of curve in methanol × 100.

Calibrations curves of mycotoxins dissolved in blank urine extract samples and in methanol were constructed at concentration levels ranging from the LOQ of each mycotoxin to 1000 μg/L.

Finally, LODs and LOQs were obtained using the criterion of a signal-to-noise ratio (S/N) of ≥3 for LOD and S/N ≥ 10 for LOQ.

### 4.5. LC-ESI-qTOF-MS Determination

An Agilent 1200-LC system (Agilent Technologies, Palo Alto, CA, USA) equipped with a vacuum degasser, autosampler, and binary pump was used for the chromatographic determination. The column used consisted of a Gemini^®^ NX-C18 (3 µM, 150 × 2 mm ID) (Phenomenex). The mobile phases were made of water (A) and acetonitrile (B), both with 0.1% of formic acid. The gradient program was as follows: 0–6 min, 50% B; 7–12 min, 100% B; 13–20 min, 50% B. The injection volume was set at 5 µL and the flow rate at 0.2 mL/min. Mass spectrometry (MS) analysis was performed employing a 6540 Agilent Ultra-High-Definition Accurate-Mass q-TOF-MS coupled to the HPLC, equipped with an Agilent Dual Jet Stream electrospray ionization (Dual AJS ESI) interface in positive and negative ionization modes under the following conditions: interface in positive and negative ionization modes; drying gas flow (N2) 12.0 L min^−1^; nebulizer pressure, 50 psi; gas drying temperature, 370 °C; capillary voltage, 3500 V; fragmentor voltage, 160 V. Analysis was carried out in MS mode and MS spectra were collected within the scan range of 50–1500 *m*/*z*. Integration and data acquisition were performed employing the Mass Hunter Workstation software.

### 4.6. Risk Assessment

Risk assessment provides an overview of the potentially hazardous exposure to mycotoxins. Risk assessment employing biomarker quantification uses the excreted levels of the contaminant in urine and aims to estimate the intake level. Molecular biomarkers of mycotoxins (mycotoxin metabolites or mycotoxin bioconjugated forms) can be used to measure human exposure.

According to Solfrizzo et al. [37], the Probable Daily Intake (PDI) can be calculated based on the results of mycotoxin biomarkers detected in urine, using the following expression:

PDI (µg kg bw/day) = C × V × 100/W × E

where C refers to the concentration of mycotoxins biomarker in urine (µg/L), V to the volume of urine excreted in 24 h, established in a mean of 1.5 L [38]. W refers to body weight (kg), established at 82 kg for males and 67.2 kg for females according to EFSA guidance [39]. Finally, E constitutes the mycotoxin excretion rate (%), calculated as approximately 50% for OTA [40], 10% for ZEA [41], and 1.3% for AFs [42].

In order to calculate mycotoxin PDIs, different exposure scenarios were considered: the mean of positive urine samples, where only positive samples were considered; and the lower bound (LB) and upper bound (UB) scenarios, where data below LOD were processed according to EFSA recommendations [43]. Thus, zero was assigned when mycotoxins were not detected or were detected below the limit of quantification in the LB scenario, while the limit of detection was assigned in the UB scenario.

Then, PDIs were compared with the established TDIs to estimate the potential risk of exposure to the mycotoxins. A tolerable weekly intake (TWI) of 0.12 µg/kg bw/week has been fixed for OTA by the EFSA CONTAM Panel [44] and a TDI of 0.25 µg/kg bw/day has been established for ZEA [45]. However, no TDI has been established for AFs, as they are considered genotoxic and carcinogenic compounds, causing hepatocellular carcinoma [46,47]. In this case, risk assessment is typically based on the margin of exposure (MoE) [48]. Moreover, for OTA, EFSA [49] concluded that the use of a health-based guidance value for OTA is no longer appropriate, but instead a margin of exposure (MoE) should be calculated for both neoplastic and non-neoplastic effects.

The MoE tool is used in risk assessment to evaluate substances that are both genotoxic and carcinogenic. MoE can be calculated using the benchmark dose lower confidence limit (BMDL), obtained from animal studies, divided by estimated PDI. Subscript 10 indicates the percentage of the confidence level of the dose response curve. Thus, MoE can be calculated following this equation:

MoE = (BMDL 10)/(intake values)

An MoE ≥ 10,000 indicates low public health risk associated with exposure to a genotoxic carcinogen [48].

An AFs BMD lower confidence limit (BMDL10) for a 10% increase in cancer incidence obtained from animal study data modeling of 170 ng/kg bw/day was proposed by EFSA [50], while a BMDL10 (14,500 ng/kg bw/day for neoplastic effects) was considered according to EFSA guidelines concerning OTA risk assessment [49]. To the best of our knowledge no BMDL10 has so far not been established for OTB.

## Figures and Tables

**Table 1:** Analytical parameters for QuEChERS extraction in urine samples: recoveries, matrix effects, limits of detection, and quantification and linearity.

Mycotoxin	Recoveries 50 μg/L± RSD (%)		Recoveries 100 μg/L ± RSD (%)		Matrix Effects (SSE %)	Limits of Detection (LODs) (μg/L)	Limits of Quantification (LOQs) (μg/L)	Linearity R^2^
	Intra-Day Analysis	Inter-Day Analysis	Intra-Day Analysis	Inter-Day Analysis				
AFB2	90 ± 20	115 ± 12	80 ± 2	98 ± 20	66	1.5	5	0.997
AFG2	72 ± 19	77 ± 20	75 ± 16	105 ± 20	86	1.5	5	0.990
OTA	55 ± 8	52 ± 13	75 ± 19	67 ± 19	21	3	10	0.994
OTB	56 ± 18	72 ± 3	93 ± 20	99 ± 17	23	3	10	0.992
ZEA	85 ± 17	108 ± 10	80 ± 4	96 ± 20	22	1.5	5	0.994
αZOL	76 ± 5	84 ± 6	84 ± 1	68 ± 20	49	1.5	5	0.994

**Table 2:** Incidences (%) and contents (µg/L) of mycotoxins detected in urine samples.

Mycotoxin	AFB2	AFG2	OTB
Incidence (%)	32	41	9
Minimum concentration (µg/L)	<LOQ	<LOQ	<LOQ
Maximum concentration (µg/L)	60.98	69.42	38.88
Mean of total samples (µg/L)	5.30	9.26	1.62
Mean of positive samples (µg/L)	16.48	23.81	18.17
Mean in male urine samples (µg/L)	19.16	24.97	38.88
Mean in female urine samples (µg/L)	14.78	22.28	12.99

**Table 3:** PDIs calculated based on the mycotoxin biomarker urinary levels among the participants.

	Mean Positive Samples		LB Scenario		UB Scenario	
Mycotoxin	Mean PDI		Mean PDI		Mean PDI	
	(µg/kg bw/day)		(µg/kg bw/day)		(µg/kg bw/day)	
	Males	Females	Males	Females	Males	Females
AFB2	23.19	28.3	7.46	9.1	8.89	10.85
AFG2	33.52	40.9	13.03	15.9	14.1	17.2
OTB	0.66	0.81	0.06	0.07	0.16	0.19
